# Supplementary material for: Comparison of the effectiveness of different normalization methods for metagenomic cross-study phenotype prediction under heterogeneity
Source: Sci Rep. 2024 Mar 25;14:7024. doi: 10.1038/s41598-024-57670-2 (PMC10963794; doi:10.1038/s41598-024-57670-2)
Supplement: Supplementary file 1 — Supplementary Information. [file 41598_2024_57670_MOESM1_ESM.pdf]

Supplementary Tables

Table S1. Characteristics of IBD datasets, including country, number of control samples (No. of control), number of CRC samples (No. of CRC), number of species in each dataset (No. of species), percentage of zero values in each dataset (zero percentage), DNA extraction kits (DNA-Exk), sequencing platforms (Seq-Plat), and reference.

Dataset	Country	No. of controls	No. of cases	No. of species	zero percentage	DNA-Exk	Seq-Plat	Reference
Hall	United States of America	74	185	508	86.1%	other/Qiagen	IlluminaMiSeq	[1]
HMP	United States of America	426	1201	585	91.2%	Chemagen	IlluminaHiSeq	[2,3]
Ijaz	United Kingdom	38	56	356	86.2%	NA	IlluminaHiSeq	[4]
Nielsen	Denmark/Spain	248	148	606	84.5%	NA	IlluminaHiSeq	[5]
Vila	Netherlands	1135	355	711	88.1%	Qiagen	IlluminaHiSeq	[6]

Supplementary Figures

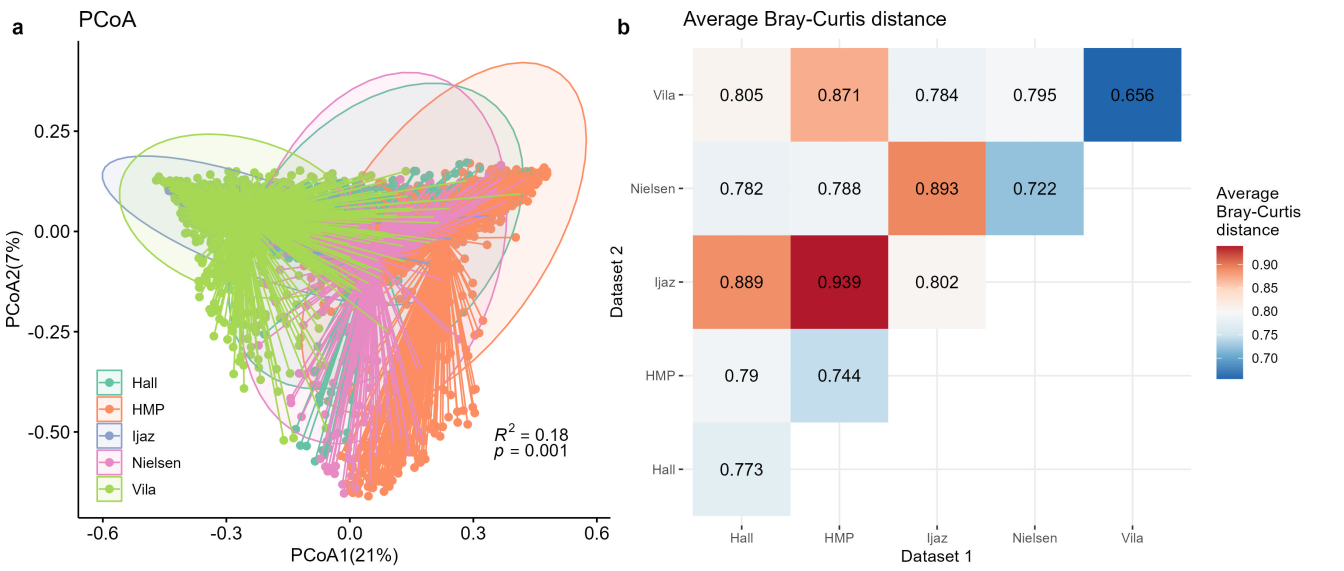

Figure S1. Different IBD populations had different background distribution patterns. (**a**) PCoA plot based on Bray-Curtis distance, with colors for different datasets. The variance explained by populations (PERMANOVA R^2) and its significance (PERMANOVA p value) were annotated in the figure. (**b**) Average Bray-Curtis distances between pairs of IBD datasets. Values on the diagonal referred to average Bray-Curtis distances between samples within the same dataset. Off-diagonal values refer to average Bray-Curtis distances between pairs of samples in different datasets. Larger values indicated a more dispersed distribution (on-diagonal) or bigger differences (off-diagonal). The figures were generated using R version 4.3.0.

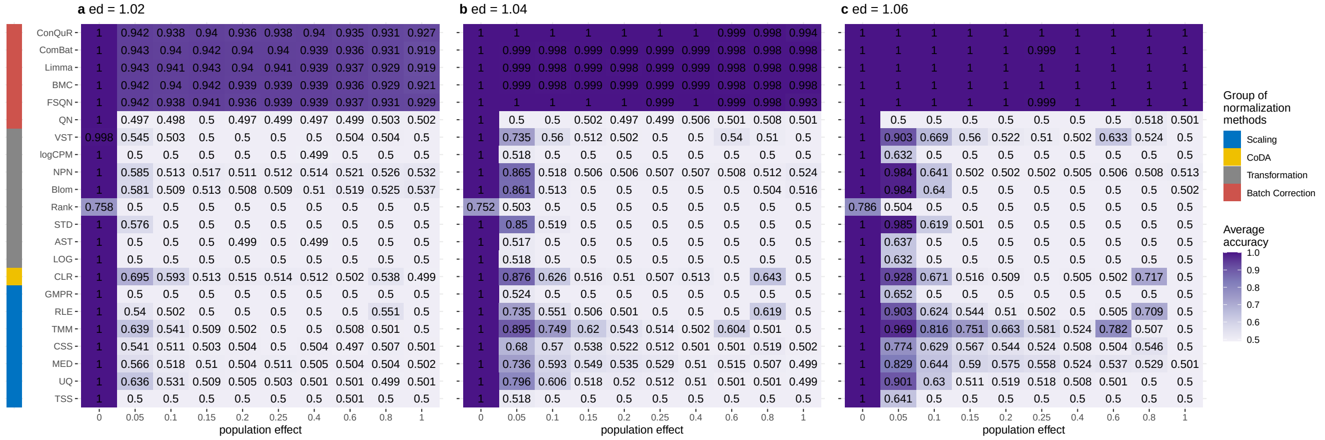

Figure S2. Heatmaps depicting average accuracy obtained from abundance profiles normalized by various methods for predicting simulated cases and controls in Scenario 1. The panels (**a**), (**b**), and (**c**) correspond to disease effects of 1.02, 1.04, and 1.06 respectively. The columns represent different values of population effects, while the rows represent different normalization methods, grouped based on their classifications in the left column. The figures were generated using R version 4.3.0.

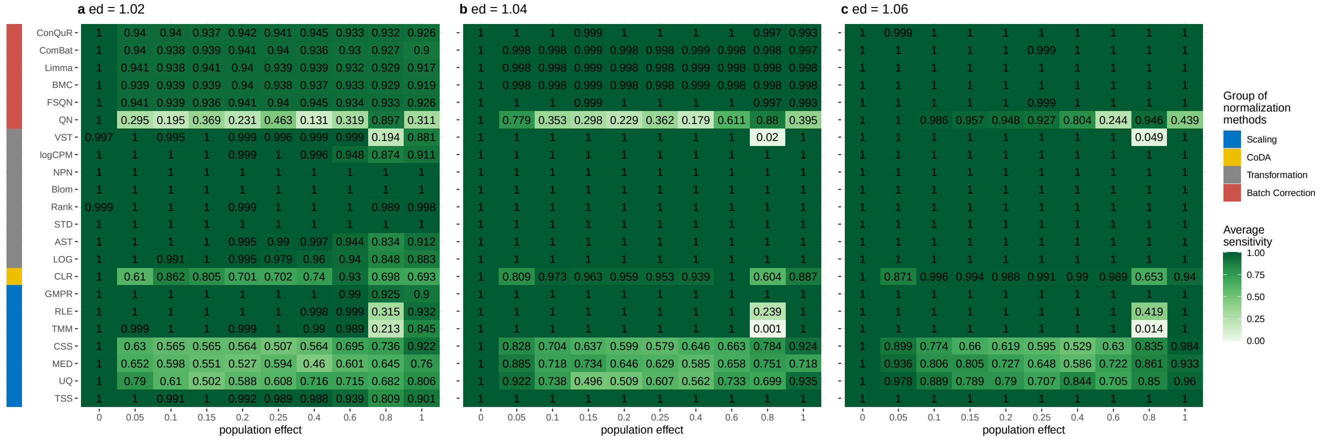

Figure S3. Heatmaps depicting average sensitivity obtained from abundance profiles normalized by various methods for predicting simulated cases and controls in Scenario 1. The panels (**a**), (**b**), and (**c**) correspond to disease effects of 1.02, 1.04, and 1.06 respectively. The columns represent different values of population effects, while the rows represent different normalization methods, grouped based on their classifications in the left column. The figures were generated using R version 4.3.0.

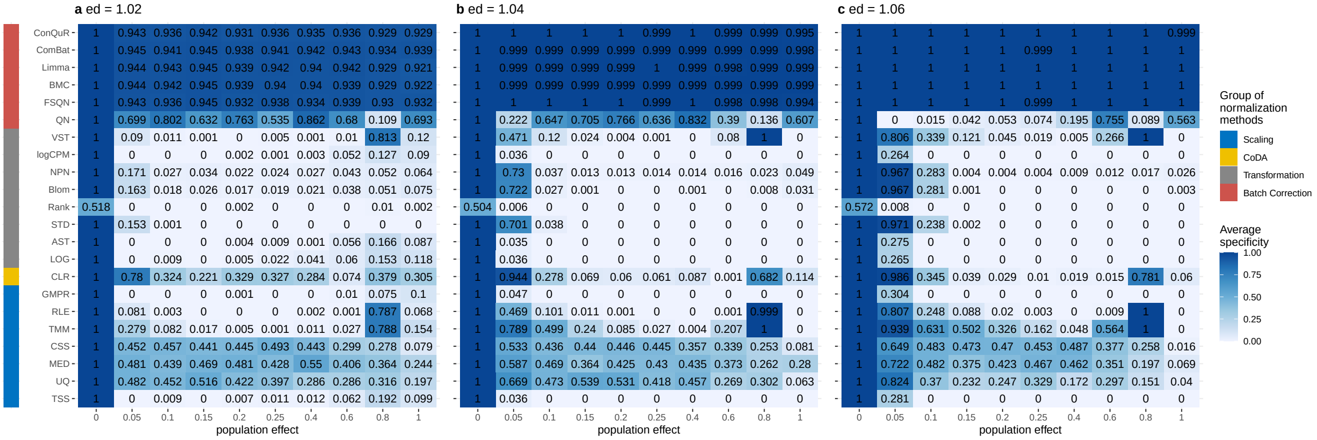

Figure S4. Heatmaps depicting average specificity obtained from abundance profiles normalized by various methods for predicting simulated cases and controls in Scenario 1. The panels (**a**), (**b**), and (**c**) correspond to disease effects of 1.02, 1.04, and 1.06 respectively. The columns represent different values of population effects, while the rows represent different normalization methods, grouped based on their classifications in the left column. The figures were generated using R version 4.3.0.

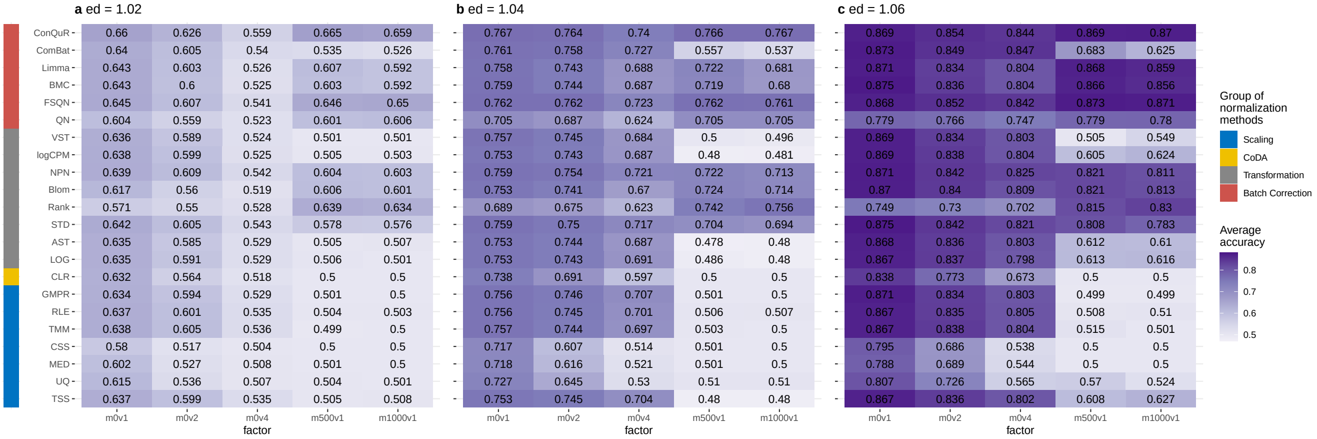

Figure S5. Heatmaps depicting average accuracy obtained from abundance profiles normalized by various methods for predicting simulated cases and controls in Scenario 2. The panels (**a**), (**b**), and (**c**) correspond to disease effects of 1.02, 1.04, and 1.06 respectively. The columns represent different combinations of batch mean and batch variation, with "m" for batch mean adjusting the mean and "v" for batch variance adjusting the variance. The rows represent different normalization methods, grouped based on their classifications in the left column. The figures were generated using R version 4.3.0.

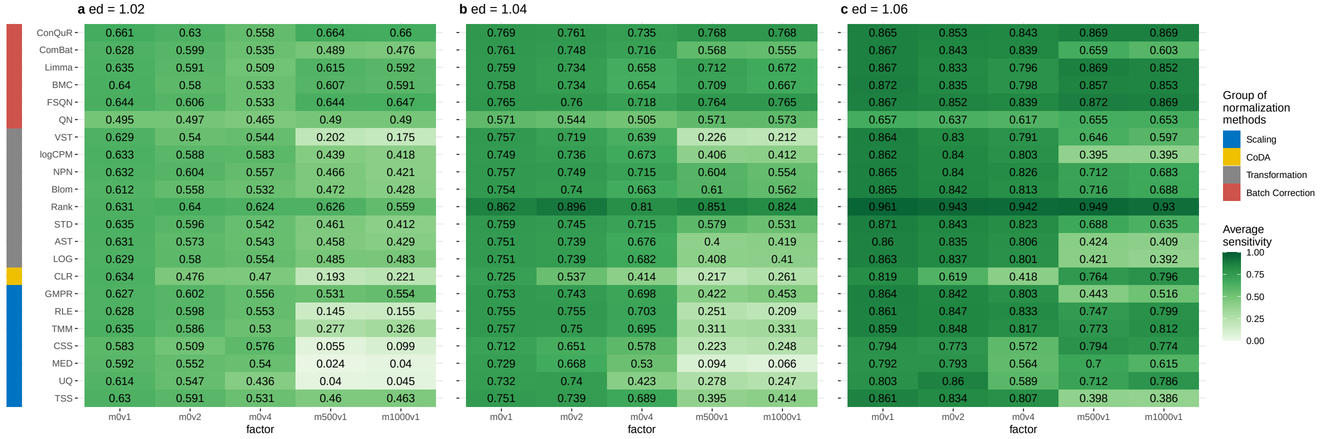

Figure S6. Heatmaps depicting average sensitivity obtained from abundance profiles normalized by various methods for predicting simulated cases and controls in Scenario 2. The panels (**a**), (**b**), and (**c**) correspond to disease effects of 1.02, 1.04, and 1.06 respectively. The columns represent different combinations of batch mean and batch variation, with "m" for batch mean adjusting the mean and "v" for batch variance adjusting the variance. The rows represent different normalization methods, grouped based on their classifications in the left column. The figures were generated using R version 4.3.0.

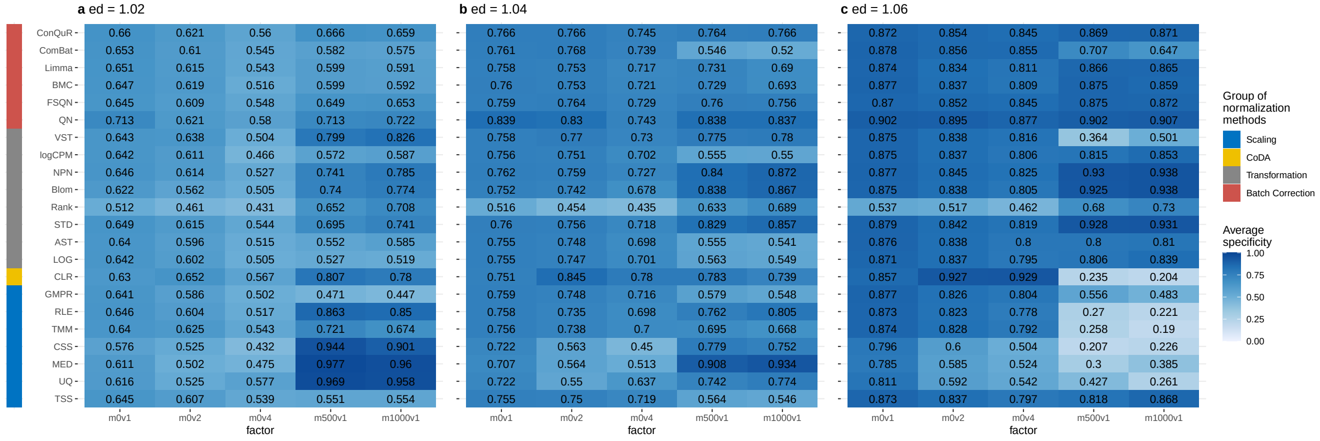

Figure S7. Heatmaps depicting average specificity obtained from abundance profiles normalized by various methods for predicting simulated cases and controls in Scenario 2. The panels (**a**), (**b**), and (**c**) correspond to disease effects of 1.02, 1.04, and 1.06 respectively. The columns represent different combinations of batch mean and batch variation, with "m" for batch mean adjusting the mean and "v" for batch variance adjusting the variance. The rows represent different normalization methods, grouped based on their classifications in the left column. The figures were generated using R version 4.3.0.

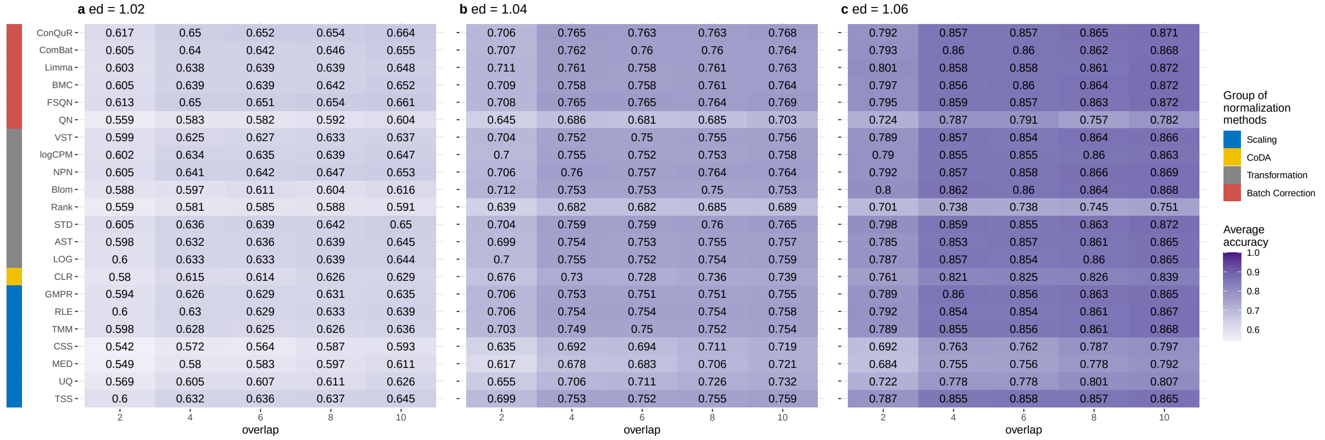

Figure S8. Heatmaps depicting average accuracy obtained from abundance profiles normalized by various methods for predicting simulated cases and controls in Scenario 3. The panels (**a**), (**b**), and (**c**) correspond to disease effects of 1.02, 1.04, and 1.06 respectively. The columns represent different numbers of overlapping disease-associated taxa in the training and testing datasets. The rows represent different normalization methods, grouped based on their classifications in the left column. The figures were generated using R version 4.3.0.

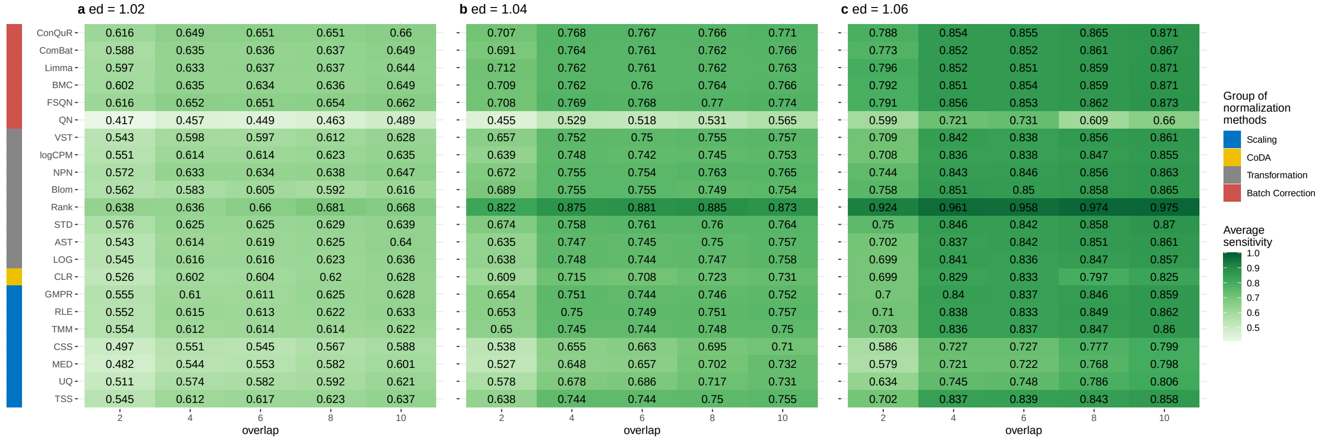

Figure S9. Heatmaps depicting average sensitivity obtained from abundance profiles normalized by various methods for predicting simulated cases and controls in Scenario 3. The panels (**a**), (**b**), and (**c**) correspond to disease effects of 1.02, 1.04, and 1.06 respectively. The columns represent different numbers of overlapping disease-associated taxa in the training and testing datasets. The rows represent different normalization methods, grouped based on their classifications in the left column. The figures were generated using R version 4.3.0.

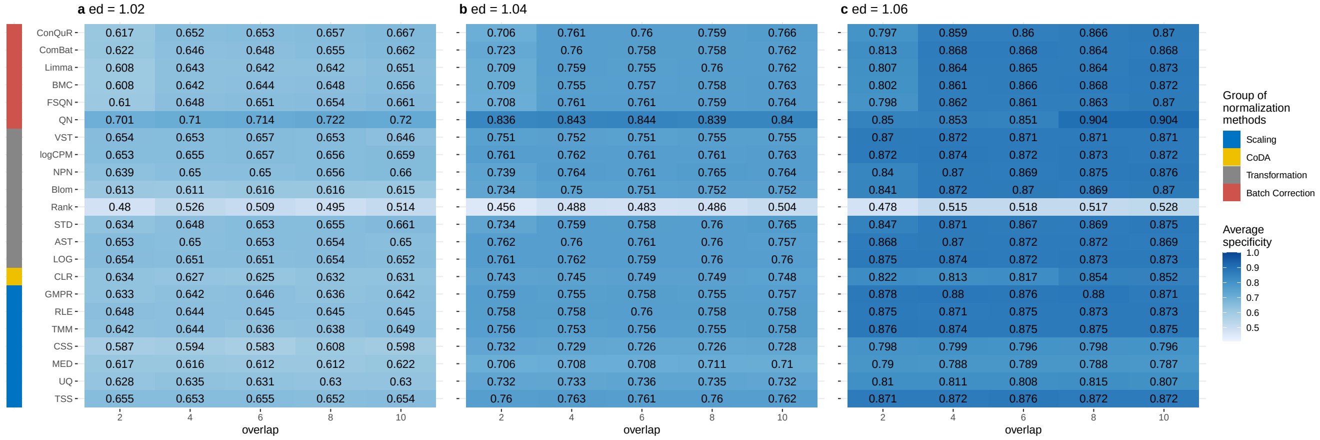

Figure S10. Heatmaps depicting average specificity obtained from abundance profiles normalized by various methods for predicting simulated cases and controls in Scenario 3. The panels (**a**), (**b**), and (**c**) correspond to disease effects of 1.02, 1.04, and 1.06 respectively. The columns represent different numbers of overlapping disease-associated taxa in the training and testing datasets. The rows represent different normalization methods, grouped based on their classifications in the left column. The figures were generated using R version 4.3.0.

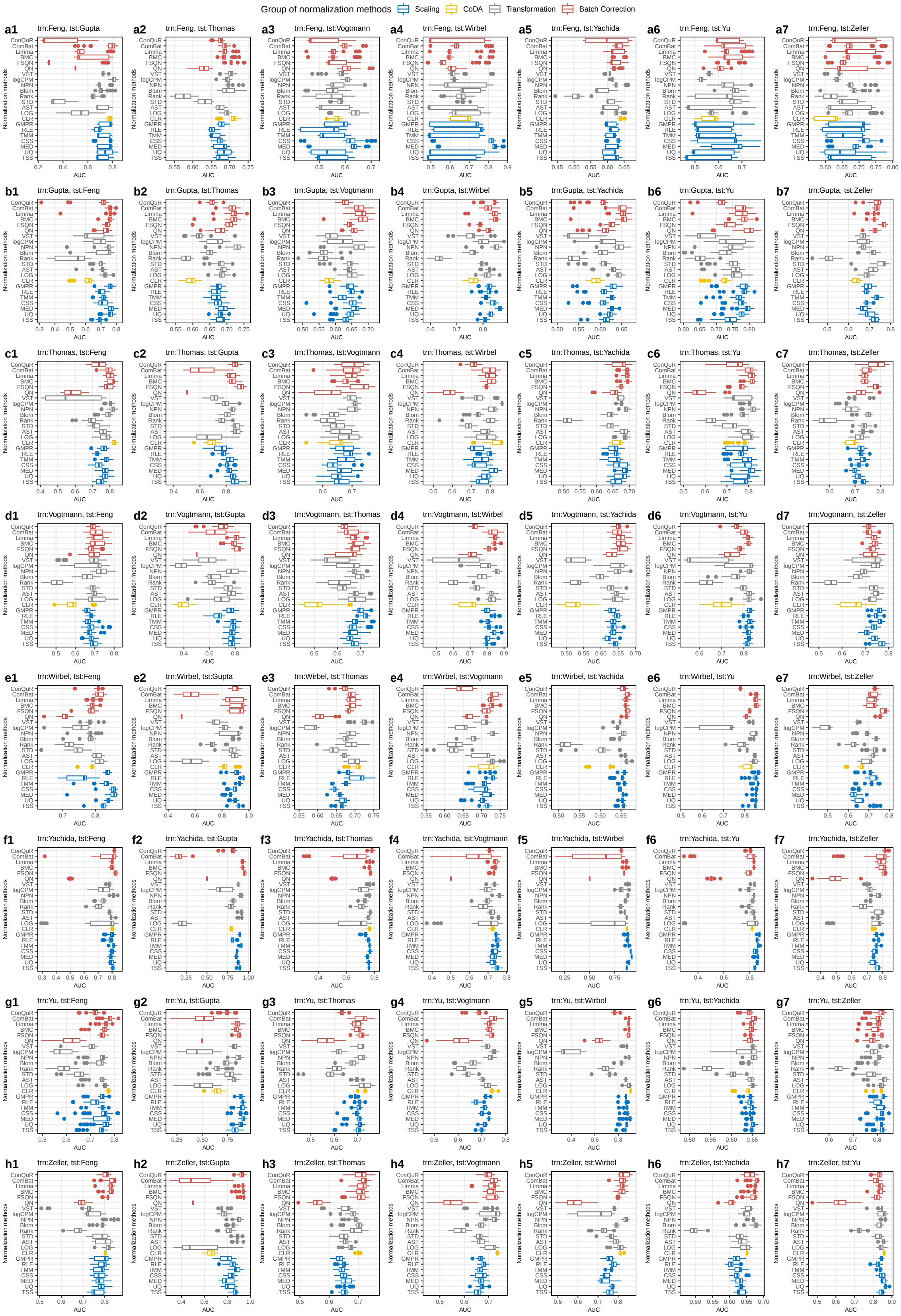

Figure S11. Box plots of AUC values over 30 repetitions in cross-dataset prediction of disease status using abundance profiles normalized by various methods on CRC datasets. The normalization methods were categorized and color-coded by their respective groups. The figures were generated using R version 4.3.0.

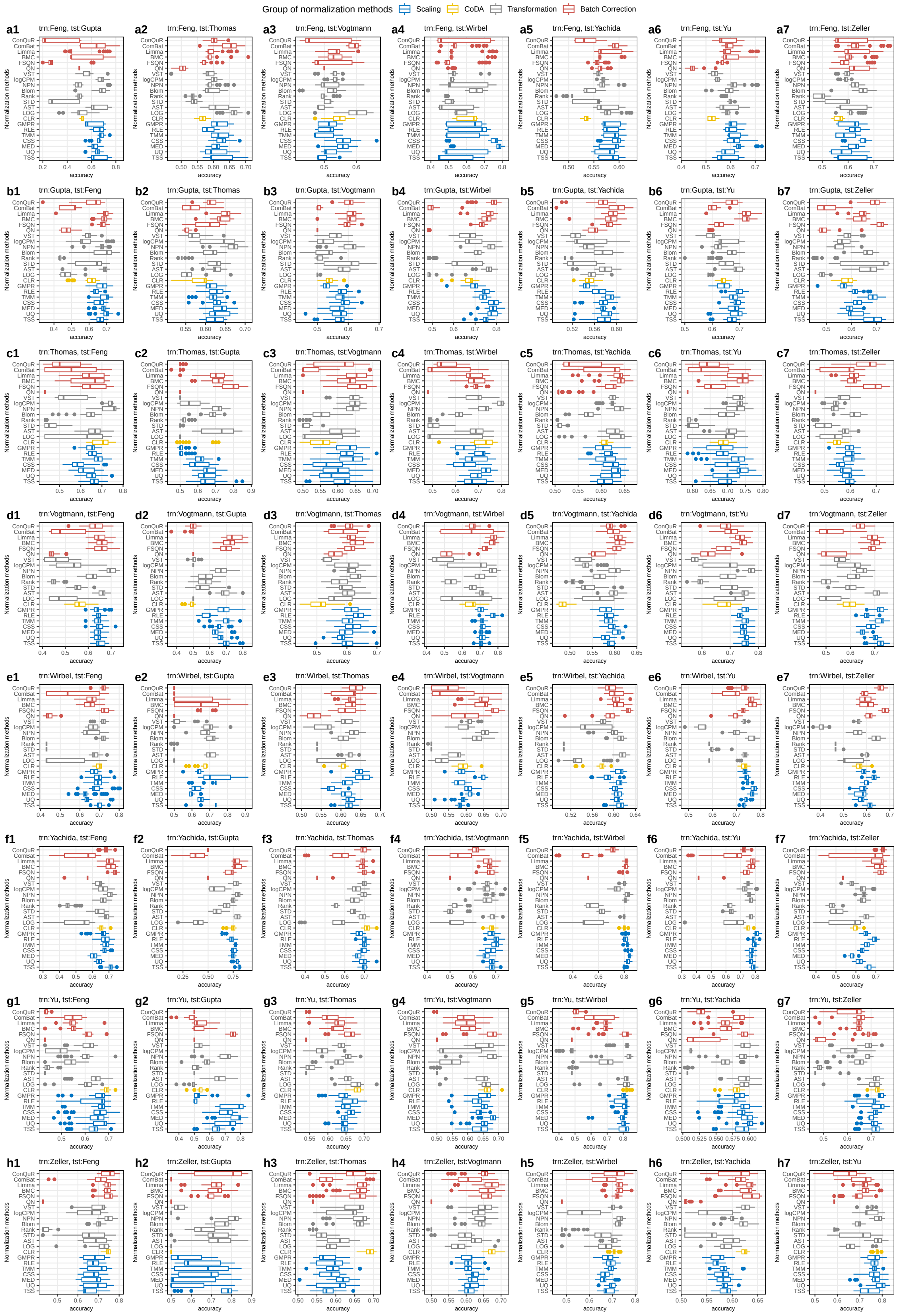

Figure S12. Box plots of accuracy over 30 repetitions in cross-dataset prediction of disease status using abundance profiles normalized by various methods on CRC datasets. The normalization methods were categorized and color-coded by their respective groups. The figures were generated using R version 4.3.0.

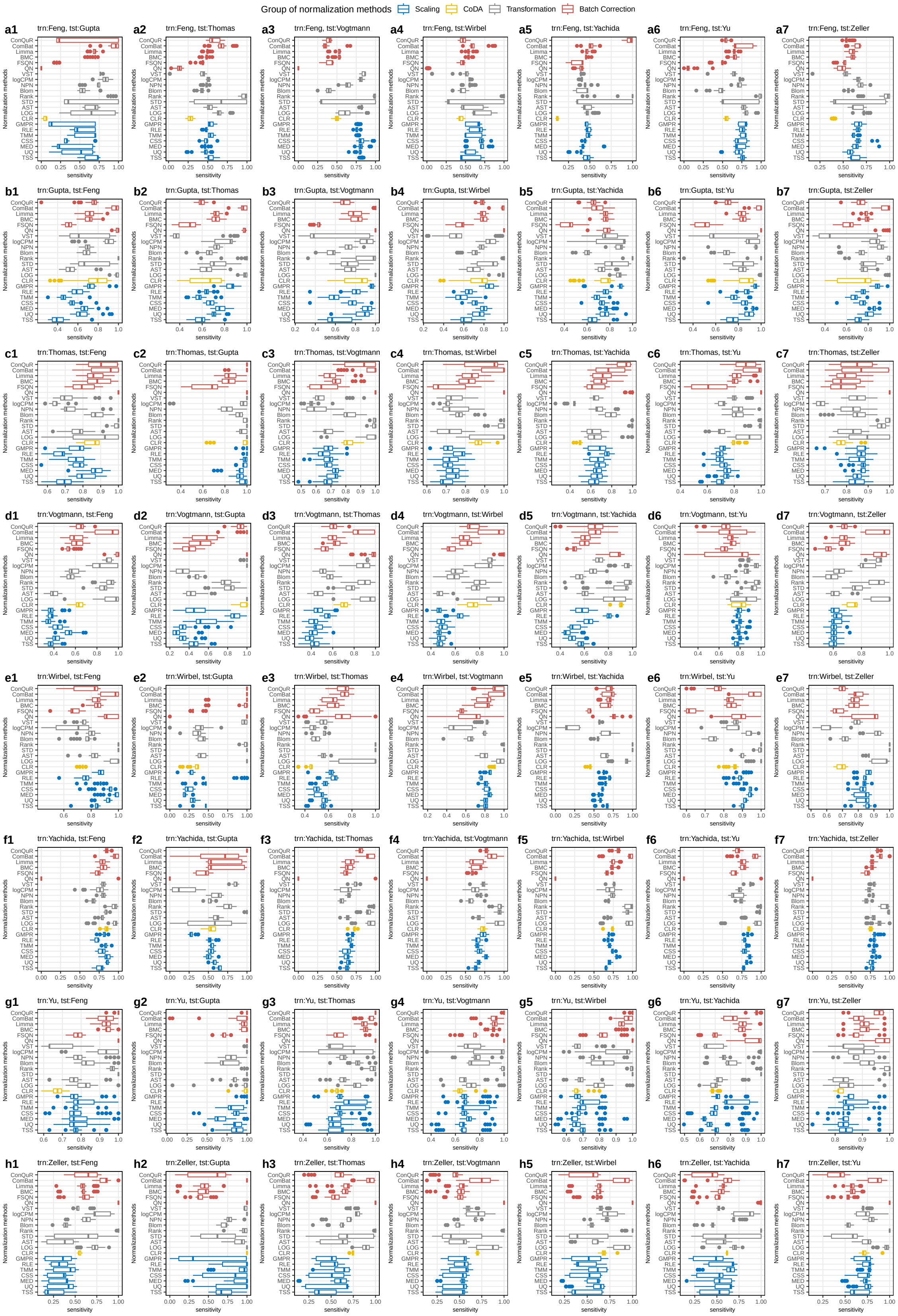

Figure S13. Box plots of sensitivity over 30 repetitions in cross-dataset prediction of disease status using abundance profiles normalized by various methods on CRC datasets. The normalization methods were categorized and color-coded by their respective groups. The figures were generated using R version 4.3.0.

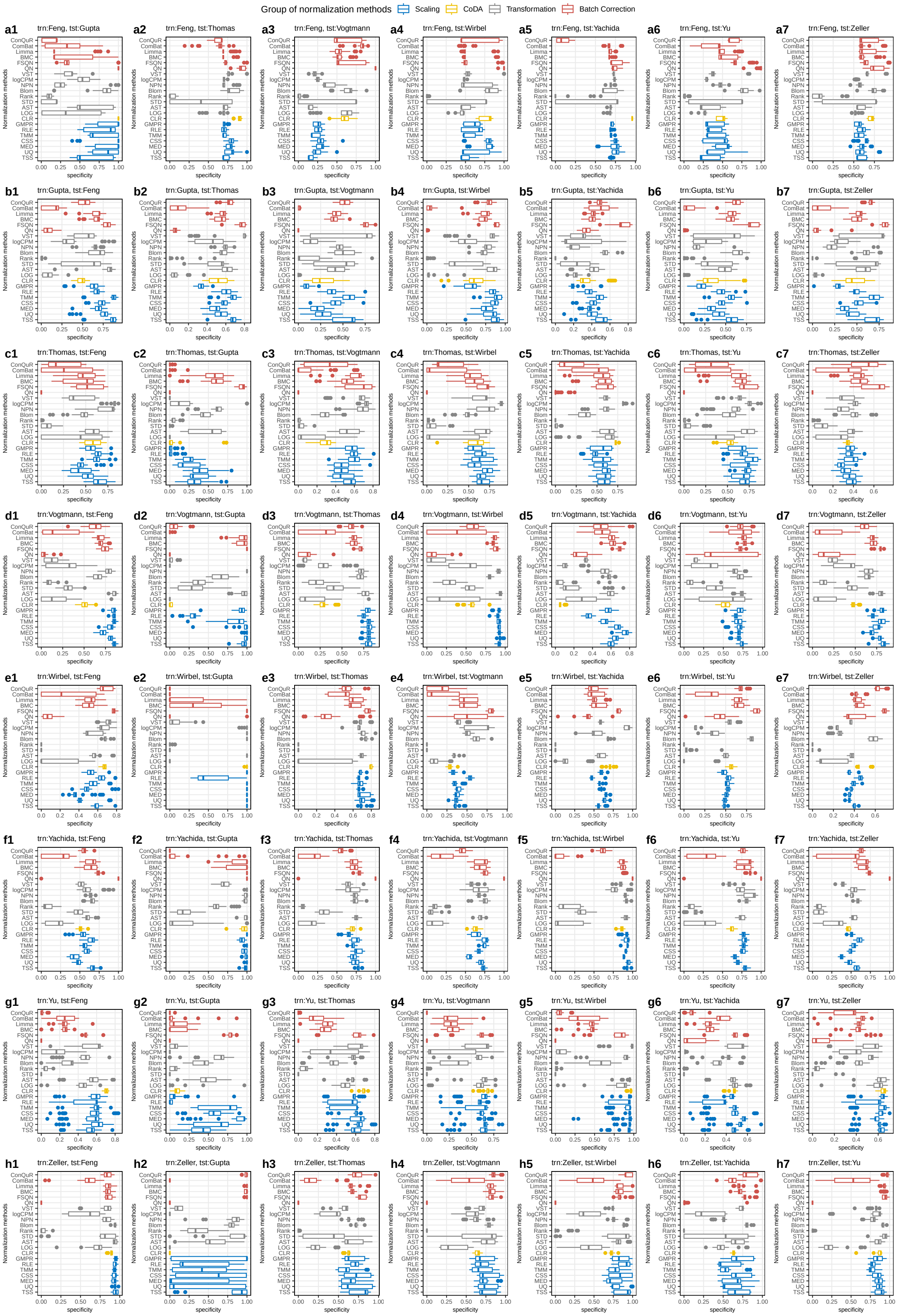

Figure S14. Box plots of specificity over 30 repetitions in cross-dataset prediction of disease status using abundance profiles normalized by various methods on CRC datasets. The normalization methods were categorized and color-coded by their respective groups. The figures were generated using R version 4.3.0.

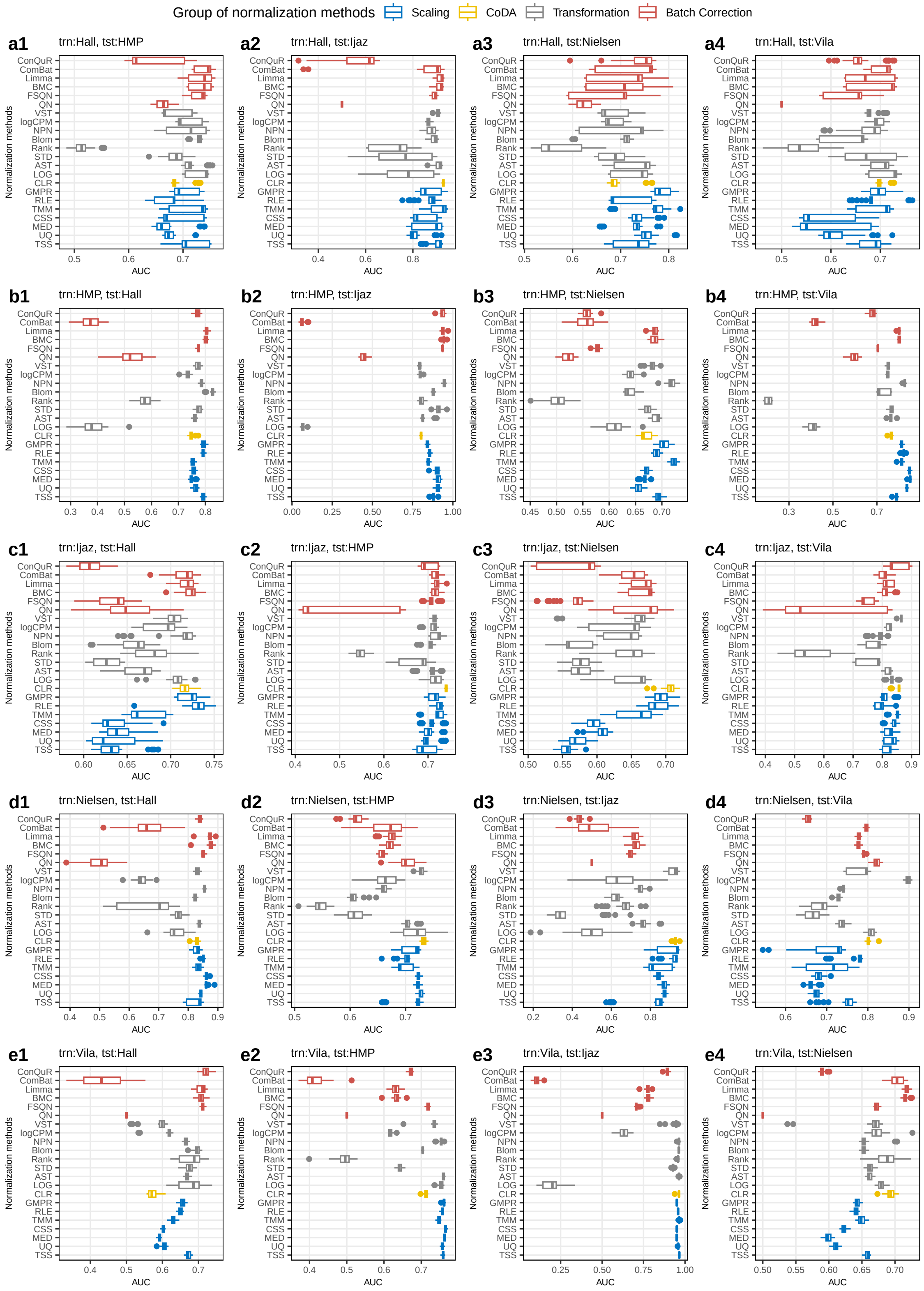

Figure S15. Box plots of AUC values over 30 repetitions in cross-dataset prediction of disease status using abundance profiles normalized by various methods on IBD datasets. The normalization methods were categorized and color-coded by their respective groups. The figures were generated using R version 4.3.0.

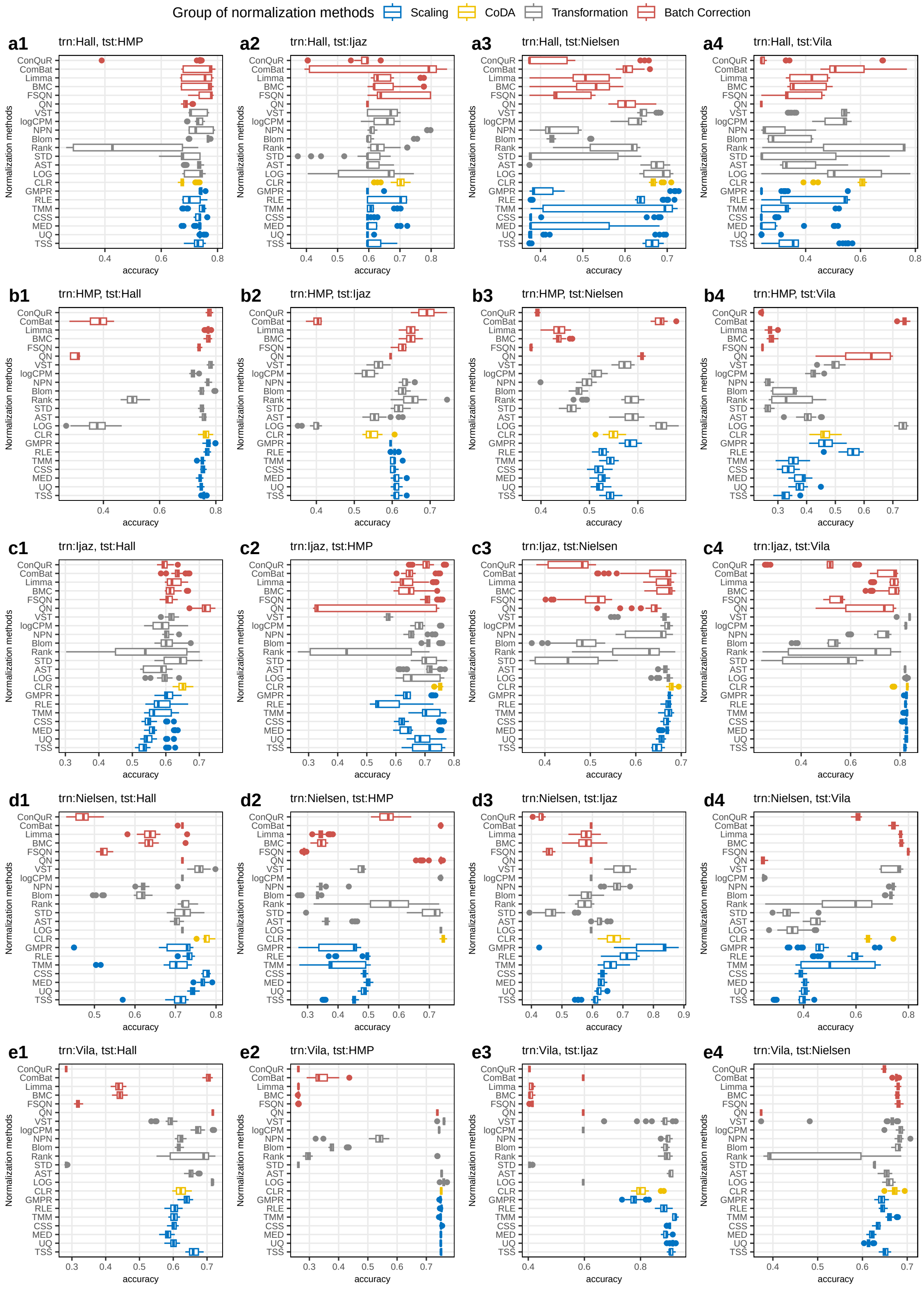

Figure S16. Box plots of accuracy over 30 repetitions in cross-dataset prediction of disease status using abundance profiles normalized by various methods on IBD datasets. The normalization methods were categorized and color-coded by their respective groups. The figures were generated using R version 4.3.0.

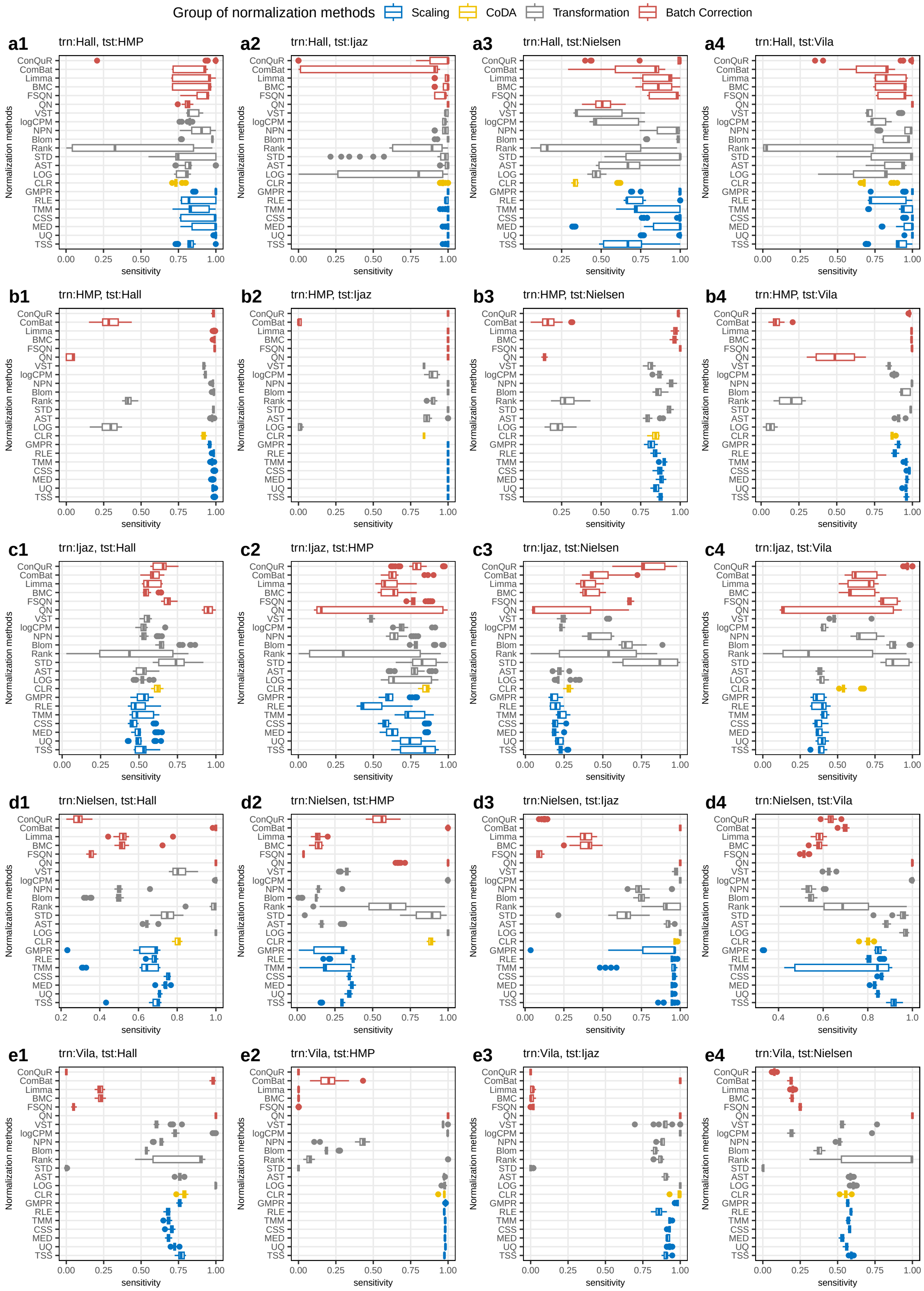

Figure S17. Box plots of sensitivity over 30 repetitions in cross-dataset prediction of disease status using abundance profiles normalized by various methods on IBD datasets. The normalization methods were categorized and color-coded by their respective groups. The figures were generated using R version 4.3.0.

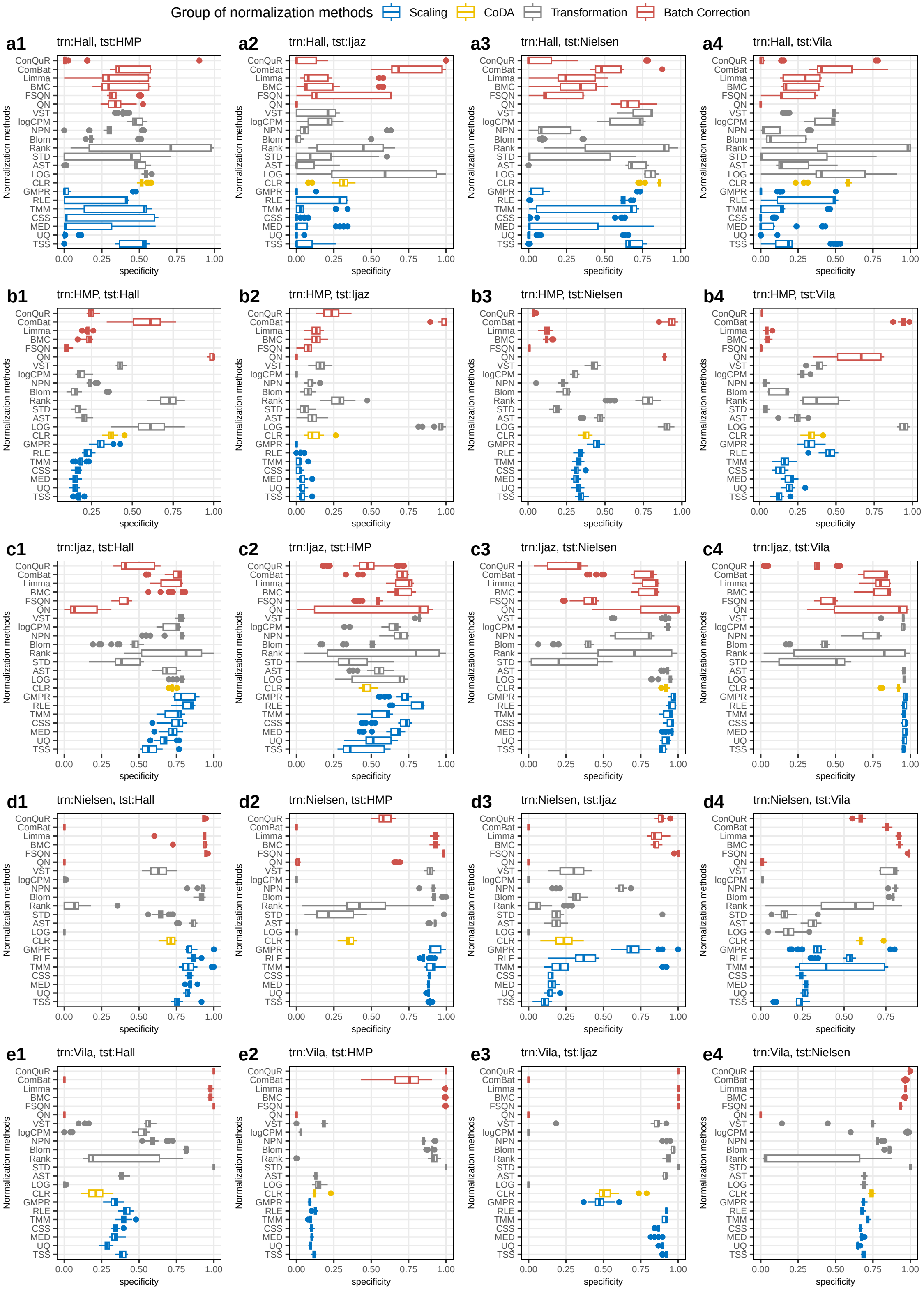

Figure S18. Box plots of specificity over 30 repetitions in cross-dataset prediction of disease status using abundance profiles normalized by various methods on IBD datasets. The normalization methods were categorized and color-coded by their respective groups. The figures were generated using R version 4.3.0.

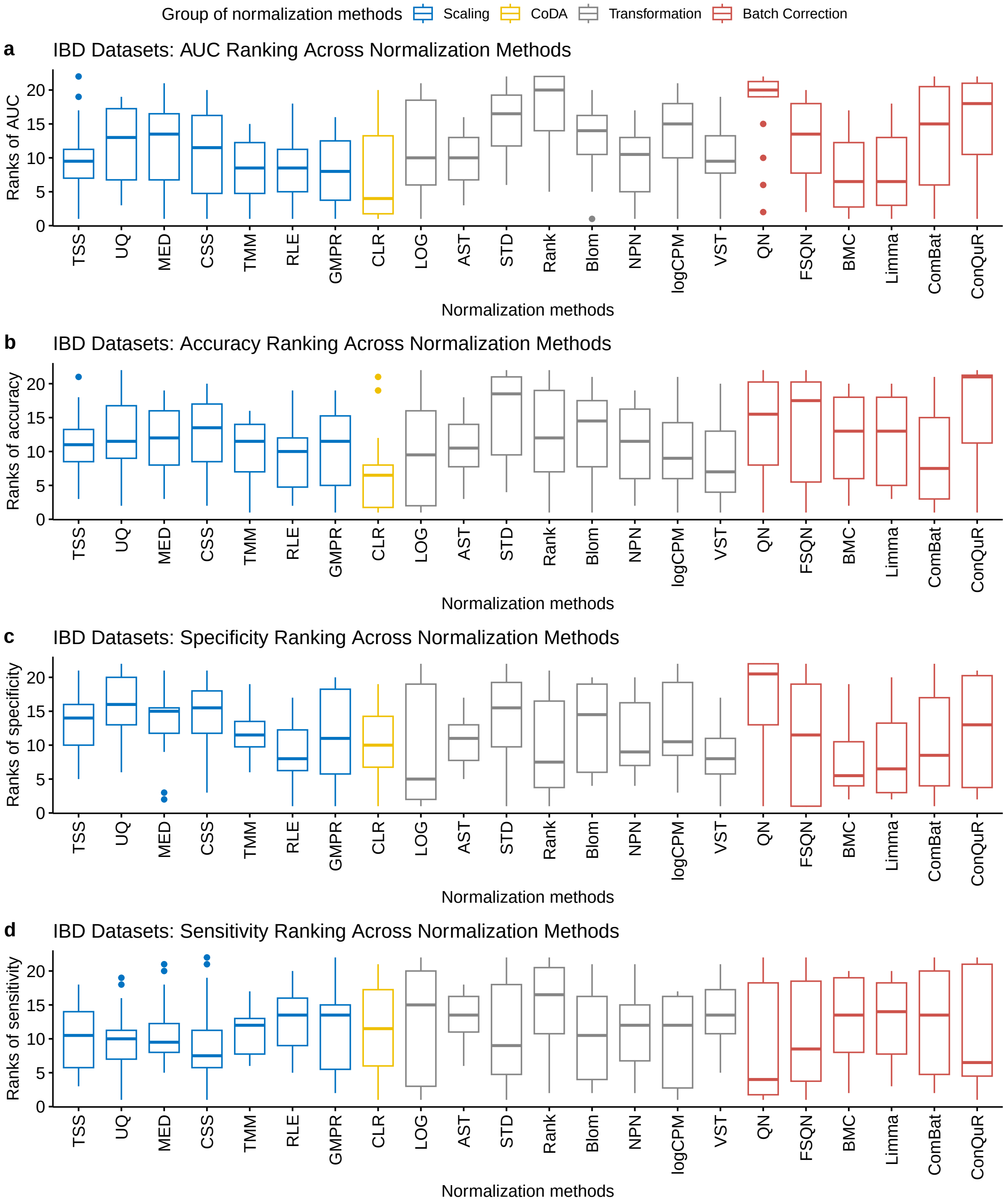

Figure S19. Distribution of ranks for 22 normalization methods in cross-dataset prediction on IBD datasets. The normalization methods are ranked based on the average AUC (**a**), average accuracy (**b**), average sensitivity (**c**), and average specificity (**d**) under the same pair of training and testing datasets. The figures were generated using R version 4.3.0.

References

1. Hall, A. B. *et al.* A novel ruminococcus gnavus clade enriched in inflammatory bowel disease patients. *Genome medicine* **9**, 103 (2017).

2. Schirmer, M. *et al.* Dynamics of metatranscription in the inflammatory bowel disease gut microbiome. *Nat. microbiology* **3**, 337–346 (2018).

3. Lloyd-Price, J. *et al.* Multi-omics of the gut microbial ecosystem in inflammatory bowel diseases. *Nature* **569**, 655–662 (2019).

4. Ijaz, U. Z. *et al.* The distinct features of microbial 'dysbiosis' of crohn's disease do not occur to the same extent in their unaffected, genetically-linked kindred. *PloS one* **12**, e0172605 (2017).

5. Nielsen, H. B. *et al.* Identification and assembly of genomes and genetic elements in complex metagenomic samples without using reference genomes. *Nat. biotechnology* **32**, 822–828 (2014).

6. Vich Vila, A. *et al.* Gut microbiota composition and functional changes in inflammatory bowel disease and irritable bowel syndrome. *Sci. translational medicine* **10**, eaap8914 (2018).
